# Managing the Unpredictable: Mechanistic Analysis and Clinical Recommendations for Lamotrigine Treatment after Bariatric Surgery

**DOI:** 10.3390/jcm10235627

**Published:** 2021-11-29

**Authors:** Daniel Porat, Carmil Azran, Hasan Kais, Arik Dahan

**Affiliations:** 1 Department of Clinical Pharmacology, School of Pharmacy, Faculty of Health Sciences, Ben-Gurion University of the Negev, P.O. Box 653, Beer-Sheva 8410501, Israel; poratdan@post.bgu.ac.il; 2 Clinical Pharmacy, Herzliya Medical Center, Herzliya 4685107, Israel; CarmilA@hmc.co.il; 3 Division of Surgery, Shamir (Assaf Harofeh) Medical Center, Zerifin, Beer-Yaakov 7033001, Israel; hk1966@gmail.com

**Keywords:** anticonvulsant, pharmacotherapy, oral drug absorption, epilepsy, metabolic surgery

## Abstract

Bariatric surgery may alter the absorption and overall bioavailability of oral drugs. Lamotrigine is a major antiepileptic and mood stabilizer, that its use after bariatric surgery has not yet been studied. In this article, we provide a thorough mechanistic analysis of the effects of bariatric surgery on multiple mechanisms important for the absorption, bioavailability and overall pharmacokinetics of lamotrigine. Attributable to its pharmacokinetic properties and drug characteristics, the use of lamotrigine after bariatric surgery may be challenging. The complex situation in which some mechanisms may lead to increased drug exposure (e.g., decreased metabolism, weight loss) while others to its decrease (e.g., hampered dissolution/solubility, decreased gastric volume), may result in lowered, unchanged, or enhanced lamotrigine plasma levels after the surgery. We conclude with a set of clinical recommendations for lamotrigine treatment after bariatric surgery, aiming to allow better patient care, and emphasizing the extra caution that needs to be taken with these patients.

## 1. Introduction

Bariatric surgery is an effective long-term treatment for severe obesity and comorbidities [1]. Quite a few short- and long-term risks, though, pose a concern among clinicians and patients [2]. One of the least discussed complications of bariatric surgery involves altered oral drug absorption and bioavailability, potentially leading to efficacy/safety issues of various orally administered medications [3,4]. A few bariatric procedures are commonly available worldwide, including sleeve gastrectomy, some variations of gastric bypass surgeries, notably Roux-en-Y gastric bypass (RYGB) and the newer one-anastomosis gastric bypass (OAGB), and more; and while each procedure is unique in the gastrointestinal anatomic changes that it brings and despite the fact that literature data is mainly available on RYGB, all of these procedures may affect oral drug pharmacokinetics [5,6,7,8,9], with clinically significant ramifications. Until recently, the literature in this field was mainly limited to case reports and small studies. Nowadays, larger clinical trials are more common [3], however, much is still unknown regarding the different variables and mechanisms that determine the overall drug exposure following the bariatric surgery [10].

Epilepsy is a serious neurological condition requiring appropriately dosed, chronic medication treatment to allow controlled and seizure-free disease. Due to this reason, therapeutic drug monitoring is a common practice with some anticonvulsants [11]. In the context of bariatric surgery, these orally administered drugs may require extra care due to possible alterations in the mechanisms responsible for their absorption and disposition in the body [12]. In fact, some of these mechanisms are associated with increased drug exposure, while others may lead to decreased plasma drug levels [13].

Lamotrigine is a major weight neutral [14] anticonvulsant and mood stabilizer, and is among the most common medications in the US and around the world, frequently prescribed among patients with obesity, both before and after bariatric surgery. Yet, various drug characteristics make lamotrigine use challenging after these operations.

In this communication, we provide a clinically relevant analysis of the important mechanisms related to lamotrigine absorption and bioavailability after bariatric surgery. This analysis should expose clinicians and pharmacologists to the many variables involved in the complex issue of pharmacokinetics and pharmacotherapy of patients after bariatric surgery, aiming for better patient-centered care.

## 2. Lamotrigine Pharmacokinetics

### 2.1. Drug Characteristics

Lamotrigine belongs to class II of the biopharmaceutical classification system [15], as it is a low solubility and high permeability drug. Lamotrigine is a weak base (pKa = 5.7) [16], thus exhibiting pH-dependent aqueous solubility. In water at 25 °C, lamotrigine solubility is only 0.2 mg/mL. The drug is lipophilic, with logP of around 2 [17].

### 2.2. Absorption and Bioavailability of the Drug

Lamotrigine is rapidly and completely absorbed upon oral administration of an immediate-release dosage form, reaching maximal plasma concentration after around 3 h post-dose and systemic bioavailability of ~100%. Food has no effect on its absorption. Lamotrigine undergoes extensive metabolism to inactive glucuronide metabolites by the uridine 5′-diphospho-glucuronosyltransferase (UDP-glucuronosyltransferase, UGT) family of enzymes. It does not undergo enterohepatic recirculation. The elimination half-life of the drug after monotherapy among healthy volunteers and epileptic patients ranges widely and depends on use of concomitant antiepileptics, with glucuronidation inducing drugs, such as phenytoin, phenobarbital or carbamazepine, reducing the elimination half-life, and inhibitors such as valproic acid increasing it [18]. Lamotrigine dose varies with indication and age, and the therapeutic window is normally between 2.5 to 15 µg/mL [19].

### 2.3. Absorption Issues after Bariatric Surgery

Several unique drug characteristics of lamotrigine may predispose it to potential absorption issues following bariatric surgery. First of all, lamotrigine is a weak base with pH-dependent solubility. After the surgery, about 80% of the stomach is removed, including significant portion of the parietal cells, increasing the gastric pH from ~1.8 to around 6.5 after OAGB [20]. This increased gastric pH following the resection of the stomach during the bariatric procedure is expected to severely hinder the dissolution of the lamotrigine dose. Additionally, the surgery involves a great decrease in stomach volume, which means that there is less fluid available to dissolve the drug dose. Shortly after the surgery, the patient may only be able to drink a few milliliters of water upon drug administration, further limiting the dissolution of the drug dose. Additionally, after the surgery, stomach motility may be hindered [21], potentially leading to hampered disintegration of the drug product and decreased dissolution of the drug dose [20].

In cases of highly lipophilic drugs, decreased bile secretion after bypass surgeries may lead to problems with their solubilization [22]. Since lamotrigine is moderately (but not highly) lipophilic, this mechanism may or may not play a significant role in its absorption.

Importantly, since only dissolved drug is able to permeate via the enterocytes and reach the systemic circulation, hampered absorption of the drug may also be expected following the surgery. To note, the stomach pH is increased more significantly after gastric bypass than after sleeve gastrectomy (about 6.5 vs. 5, respectively), so absorption problems of lamotrigine may be especially severe after bypass procedures in particular [20]. Additionally, in bypass surgeries the duodenum and proximal jejunum are bypassed, shortening the remaining small intestine surface area and transit time available for absorption; for many drugs, and lamotrigine included, this decreased transit time throughout the small intestine may hamper the overall absorption of the drug. As for active transporter-mediated permeation, depending on the expression level of the relevant transporter in the bypassed intestinal segment, bypass surgeries may result in lower exposure of the drug to these transporters. Publications in the literature report that lamotrigine is subjected to both influx and efflux transporters, with unclear clinical impact [23,24]. It is likely that transporters have limited on lamotrigine absorption, so this mechanism does not significantly alter the overall exposure of lamotrigine.

### 2.4. Distribution Issues after Bariatric Surgery

Following bariatric surgery, great weight loss is often achieved shortly after the procedure. In addition, the patient loses fat tissue, and for lipophilic agents such as lamotrigine, this may lead to more drug remaining in the central compartment and not going to periphery, thus increasing the plasma levels of the drug, and making it available for central nervous system penetration, from where it exerts its therapeutic effect [25].

### 2.5. Metabolism Issues after Bariatric Surgery

As mentioned, lamotrigine undergoes extensive phase II metabolism to glucuronide metabolites. Importantly, it was shown that glucuronidation is enhances in obesity and decreased after bariatric surgery [26]. This phenomenon was approved in the cases of morphine and acetaminophen, both prototypical substrates for glucuronidation with decreased metabolism after surgery and high parent drug levels [27,28]. This may also be the case upon post-bariatric lamotrigine therapy. In fact, bodyweight in general, was found to be the most significant covariate on lamotrigine clearance, explained by correlation between the size of the excreting organ and bodyweight [29].

### 2.6. Excretion Issues after Bariatric Surgery

While lamotrigine is mainly eliminated via hepatic glucuronidation, decreased renal function was found to correlate with decreased lamotrigine clearance [29]. Meanwhile, while debatable, recent publications report potentially improved renal function shortly after bariatric surgery in patients with chronic kidney disease (CKD) [30,31,32]. Hence, among patients with CKD in the first year following the surgery, improved kidney function may contribute to overall decreased lamotrigine levels.

### 2.7. Summary of the Mechanistic Analysis

In this section, we provided several different mechanisms by which bariatric surgery may alter the disposition of lamotrigine. Notably, some of these mechanisms support increased drug levels after (vs. before) the surgery, while others may be responsible for decreased postoperative drug levels. Thus, high interpatient variability may be witnessed regarding the effect of the surgery on lamotrigine, with increased, decreased or unchanged pharmacokinetics are all possible. In addition, given the analysis above, the exact type of bariatric procedure that the patient undergoes may also play a significant role. Table 1 summarized the proposed mechanisms, dividing them to supporting increase vs. decrease in lamotrigine levels after the surgery.

## 3. Discussion

Increased, decreased or unchanged lamotrigine levels after bariatric surgery are all possible, given the presence of factors influenced by the surgery, that promote increased drug levels, as well as factors that promote decreased levels. The overall effect of the surgery may depend on the individual patient characteristics [33,34], such as concomitant drugs taken, as well as the specific bariatric procedure undergone. In addition, when two or more altered mechanisms are involved, the magnitude and direction of changes in lamotrigine pharmacokinetics may vary [35], and while the most dominant mechanism [36] may dictate the overall trend towards increased or decreased drug levels [37], this dominant mechanism may be different from patient to patient.

Similar mechanistic analysis should be performed for more drugs [38], and in vitro, in vivo and in silico models [39] should support this mechanistic approach, producing more valuable data. The aim of this mechanistic approach is to allow prediction of the pharmacokinetic changes of a given drug before prescribing it to the post-bariatric patient and design a tailored treatment plan, hence choosing the most appropriate drug and dosing regimen.

While each drug is unique in its physicochemical and pharmacokinetic properties, various drugs share at least some common features with lamotrigine. Many newly discovered drugs have moderate-to-high lipophilicity and low-to-marginal water solubility [40], so concerns related to decreased stomach and fluid volume after the surgery apply to these drugs as well. Additionally, drugs with high maximal dose (generally over 100 mg) are more challenging in this aspect. Some therapeutic classes characterized by high dose drugs are antiepileptics, antipsychotics, antibacterials, antivirals and nonsteroidal anti-inflammatory drugs (NSAIDs). Among the drugs with basic functional group, likely to exhibit decreased solubility after gastrectomy, similarly to lamotrigine, are antipsychotics, antidepressants, antivirals, antifungals, alpha and beta-blockers, anti-anxiety medications and oral anticancer agents such as tyrosine kinase inhibitors, and more. Other drugs undergoing glucuronidation as a major metabolic pathway include (but not limited to) morphine, acetaminophen, lorazepam, mycophenolic acid, valproic acid and olanzapine, and this may support potential decreased metabolism and consequent increased drug exposure.

Realizing the need for extra care when prescribing to a patient after bariatric surgery is of importance, given the unpredictable effects of the surgery. While only older antiepileptics are routinely monitored, therapeutic drug monitoring of newer antiepileptics is also warranted after the surgery [12]. Indeed, lamotrigine should be monitored, both for potentially altered trough plasma drug levels and for clinical signs of safety issues/treatment failure. For rational management of such cases, recommendations for lamotrigine treatment after bariatric surgery are provided in the following section.

## 4. Clinical Recommendations

In this section we wish to summarize practical considerations for lamotrigine treatment of patients after bariatric surgery. Periodic therapeutic drug monitoring is important after the surgery. Especially in the first few months after bariatric surgery, lamotrigine levels should be checked frequently. Preferably, lamotrigine blood levels can also be measured shortly before the surgery to discern basal effective levels that should then be aimed after the operation [12]. Involvement of clinical pharmacists as advisors in the drug treatment is beneficial for both surgeons and patients.

Immediately after the surgery, the patient should be moved to dispersible tablets or liquid dosage form, or alternatively, should crush their immediate-release tablets and spread the powder in food/drink prior to ingestion (according to package insert or available company data). This is especially indicated in cases hampered drug dissolution/disintegration is likely. It is worthwhile to note that caution should be taken with extended-release dosage forms because oftentimes they should not be crushed. In case a liquid dosage form is to be used, it is important to make sure that it does not contain non-absorbable sugars, in light of the risk for dumping syndrome. In cases drug levels drop after the surgery, gradual dose increase is, of course, an option. A specific consideration regarding bariatric surgery, may be to split the daily dose; for instance, if the patient is taking lamotrigine 200 mg once daily, shifting to 100 mg twice daily may aid to prevent hampered dissolution of the drug dose. On the other hand, if drug levels are found to be high and/or the patient cannot tolerate the lamotrigine treatment, dose may have to eventually be gradually decreased. To note, some of these undesirable situations may be temporary, as adaptation mechanisms may occur in the months following the surgery, and changes in weight also take place [41].

If lamotrigine treatment is not tolerated or is ineffective, a second antiseizure medication should be added and lamotrigine may be tapered down, if appropriate, only after the new drug reaches steady state levels [42]. Importantly, some antiepileptics are glucuronidation inducers (phenytoin, phenobarbital and carbamazepine) or inhibitors (sodium valproate), so a pharmacokinetic drug-drug interaction may affect lamotrigine levels. Among the bariatric patients, over 75% are females, and the average age of the operated patient is 42, so many of these patients are of childbearing potential [43]. Therefore, while valproate (along with lamotrigine) is a first line anticonvulsant for various different epileptic disorders, in many bariatric patients, valproate treatment should not be initiated in case of unsatisfactory lamotrigine treatment following the surgery, due to being highly teratogenic [42]. Among the other antiepileptic drugs, levetiracetam and topiramate are less likely to be affected by the bariatric surgery given their physicochemical properties and pharmacokinetic profiles, and thus may seem attractive if lamotrigine treatment is not effective/tolerated after surgery [12]. Yet, an alternative/adjuvant drug should be chosen primarily based on the specific epileptic syndrome of the patient. In addition, topiramate has less favorable side-effect profile than other alternatives [42]. Alternatively, non-oral dosage forms can be used at least temporarily, immediately after the surgery. This will sidestep the unpredictable outcomes of oral drug administering after bariatric surgery.

## 5. Conclusions

This article shows the complexity of drug treatment after bariatric surgery [44]. Consultation with a clinical pharmacist that specializes in drug therapy after bariatric surgery is necessary to allow safe and effective drug treatment in these patients. Lamotrigine, although not routinely monitored compared to other antiepileptic drugs, should be closely monitored soon after and at least a year following the bariatric surgery.

## Figures and Tables

**Table 1:** Summary of mechanisms involved in increased (↑) or decreased (↓) lamotrigine levels after gastric bypass or sleeve gastrectomy surgeries. (?) is added if mechanism effects are suspected or unknown.

The Proposed Mechanism	Gastric Bypass Surgery	Sleeve Gastrectomy
**Smaller gastric volume**	↓	↓
**Increased stomach pH**	↓↓	↓
**Decreased gastric motility**	↓	↓
**Effects on bile secretions**	↓?	-
**Effects on absorption surface area**	↓	-
**Decreased exposure to carrier proteins**	?	-
**Decreased patient weight and fat tissue**	↑	↑
**Decreased metabolism (glucuronidation)**	↑	↑
**Effects on renal clearance**	↓?	↓?
